# Re-Evaluations of Zr-DFO Complex Coordination Chemistry for the Estimation of Radiochemical Yields and Chelator-to-Antibody Ratios of ^89^Zr Immune-PET Tracers

**DOI:** 10.3390/molecules26164977

**Published:** 2021-08-17

**Authors:** Ryota Imura, Hiroyuki Ida, Ichiro Sasaki, Noriko S. Ishioka, Shigeki Watanabe

**Affiliations:** 1 Research Center for Advanced Science and Technology, The University of Tokyo, 4-6-1 Komaba, Meguro-ku, Tokyo 153-8904, Japan; imura-ryota@ric.u-tokyo.ac.jp; 2 JFE Engineering Corporation, 2-1 Suehiro-cho, Tsurumi-ku, Yokohama 230-8611, Japan; ida-hiroyuki@jfe-eng.co.jp; 3 Department of Radiation-Applied Biology Research, Takasaki Advanced Radiation Research Institute, National Institute of Quantum and Radiological Science and Technology (QST), 1233 Watanuki, Takasaki, Gunma 370-1292, Japan; sasaki.ichiro@qst.go.jp (I.S.); ishioka.noriko@qst.go.jp (N.S.I.)

**Keywords:** ^89^Zr, immune-PET, deferoxamine, Zr-DFO complex, antibody labeling, coordination chemistry, radioimmunoconjugate, theranostics

## Abstract

(1) Background: Deferoxamine B (DFO) is the most widely used chelator for labeling of zirconium-89 (^89^Zr) to monoclonal antibody (mAb). Despite the remarkable developments of the clinical ^89^Zr-immuno-PET, chemical species and stability constants of the Zr-DFO complexes remain controversial. The aim of this study was to re-evaluate their stability constants by identifying species of Zr-DFO complexes and demonstrate that the stability constants can estimate radiochemical yield (RCY) and chelator-to-antibody ratio (CAR). (2) Methods: Zr-DFO species were determined by UV and ESI-MS spectroscopy. Stability constants and speciation of the Zr-DFO complex were redetermined by potentiometric titration. Complexation inhibition of Zr-DFO by residual impurities was investigated by competition titration. (3) Results: Unknown species, ZrH*_q_*DFO_2_, were successfully detected by nano-ESI-Q-MS analysis. We revealed that a dominant specie under radiolabeling condition (pH 7) was ZrHDFO, and its stability constant (logβ_111_) was 49.1 ± 0.3. Competition titration revealed that residual oxalate inhibits Zr-DFO complex formation. RCYs in different oxalate concentration (0.1 and 0.04 mol/L) were estimated to be 86% and >99%, which was in good agreement with reported results (87%, 97%). (4) Conclusion: This study succeeded in obtaining accurate stability constants of Zr-DFO complexes and estimating RCY and CAR from accurate stability constants established in this study.

## 1. Introduction

**Figure 1:**
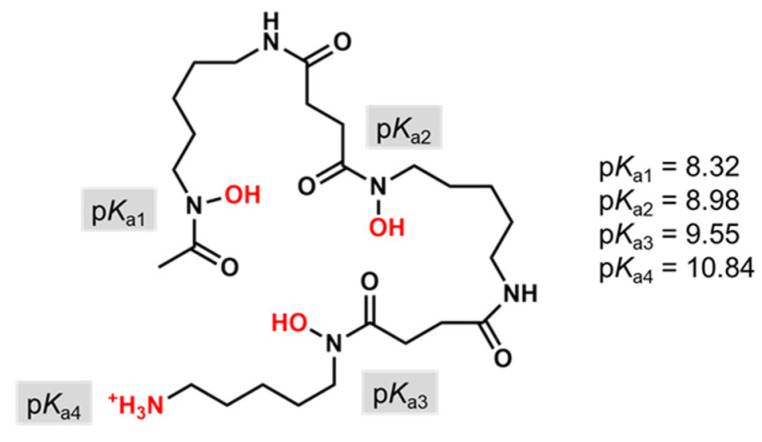
Chemical structure and p*K*_a_ values of H_4_DFO. Deprotonation sites are highlighted in red.

Coordination chemistry has played an important role in evaluating the functions of radiometal-labeled radiopharmaceuticals for both diagnostic and therapeutic applications. Especially, stability constants between radiometals and bifunctional chelators (BFC) have been key parameters for the evaluation of metal ion selectivity, in-vivo stability, and the pharmacokinetics of radiopharmaceuticals. Furthermore, we can infer that stability constants obtained from carefully considered complex models can contribute to the significant parameters for the development of radiometallic radiopharmaceuticals, the radiochemical yield (RCY) in radiolabeling, and chelator-to-antibody ratio (CAR). The stability constants of refined radiometal-BFC complex models thus have great significance in the development of radiometal-labeled radiopharmaceuticals. Interest in zirconium-89 (^89^Zr) for immuno-positron emission tomography (immuno-PET) has been accelerating in recent years due to its favorable decay characteristics: a suitable half-life (78.4 h) for antibodies that take a few days to reach the tumor and a low positron (β^+^) energy (395 keV), which results in good PET image resolution [1,2,3,4,5]. These characteristics have encouraged the development of ^89^Zr-labeled monoclonal antibodies (mAb) as ^89^Zr-immunePET reagents for theranostic applications to therapeutic radiometal-labeled antibodies, such as ^177^Lu. The naturally occurring hydroxamate-type siderophore, deferoxamine B (DFO), a small molecular iron chelator, is the main BFC used with these ^89^Zr-mAbs because it can strongly bind ^89^Zr at room temperature in high radiochemical yield (RCY) [6]. Physicochemical properties, including thermodynamics data, have been reported on the complex of common chelating agents and Zr [7,8]. However, despite the focused development of ^89^Zr-DFO-mAbs, there are only a few studies describing the coordination chemistry of Zr-DFO complexes. DFO is composed of alternating 1,5-diaminopentate and succinic acid residues, as shown in Figure 1, and an H_4_DFO^+^ ligand with p*K*_a_ values (25 °C, *I* = 0.1) of 8.32, 8.98, and 9.55 for the three hydroxamic acids and 10.84 for the protonated pendant amine [9].

Summers et al. recently proposed that volatilized Zr-DFO complex salt forms 8-coordinated Zr with oxygen ligands, and the chemical formula should be Zr(DFO)(OH)_2_ as the results of an extended X-ray absorption fine structure (EXAFS) study [10]. On the other hand, the complex structure of Zr-DFO in aqueous solution and their stability constants are also important for RCY evaluation ^89^Zr-DFO-mAb. The stability constants of the Zr-DFO complex are defined by the following equation (charges are omitted for clarity):

(1) $$\beta_{pqr}=\frac{\left[{\mathrm{Zr}}_{p}H_{q}{\mathrm{DFO}}_{r}\right]}{{\left[\mathrm{Zr}\right]}^{p}{\left[H\right]}^{q}{\left[\mathrm{DFO}\right]}^{r}}(p\geq 1,\;4r>q\geq 0,r\geq 1)$$

**Scheme 1:**
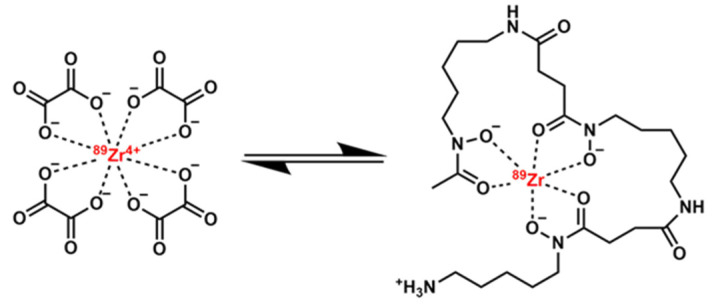
Reaction between Zr(ox)_4_ and ZrHDFO. This ligand exchange equilibrium is expected around [DFO]_T_ = 10^−6^ mol/L.

Savastano et al. determined that the main Zr-DFO species and their stability constants were ZrHDFO (logβ_111_ = 36.14) and Zr_2_H_5_DFO_3_ (logβ_253_ = 134.1) by potentiometric titration experiments [11]. Meanwhile, Toporivska et al. reported logβ_111_ was 46.4–47.7 by potentiometric and competition UV-vis titration experiments. The reported values in the two literatures differ by the order of 10^10^ [12]. In addition, previous studies did not consider the inhibition of the formation of the Zr-DFO complex by oxalate (ox^2−^) despite the fact that oxalate strongly binds to ^89^Zr [13]. It is important to take into account the complexation of oxalate with ^89^Zr because ^89^Zr is usually prepared as oxalic acid solution in the purification process. Zr-DFO complex is expected to be formed from Zr oxalate by ligand exchange as shown in Scheme 1. In fact, the RCY estimate using the reported stability constants of Zr-DFO species and Zr(ox)_4_ (10^29.7^) was 0%, which was quite different from that obtained experimentally (87%) [14]. We therefore hypothesized that the disagreement between its estimated and experimental values resulted from the presence of one or more unknown Zr-DFO species. The aim of the present study was therefore to re-evaluate the stability constants and speciation of the Zr-DFO complexes and then to show the role of the more precise stability constants in predicting RCYs and CARs. Namely, this article describes results for stoichiometry and unknown species of the Zr-DFO complex investigated by MS spectroscopies. Although speciation of Zr-DFO complexes was determined in previous studies [11,12], it is likely that unstable species of the Zr-DFO complex were missed because they were decomposed in ionization process of the MS spectroscopies. We therefore hypothesized that unknown species could be discovered by adopting a softer ionization method than previously reported. The p*K*_a_ values, stability constants, and speciation curves were evaluated by potentiometric titration, and speciation curves were determined from the obtained parameters. Competition titration studies were also performed to reveal the species that inhibit Zr-DFO complex formation. Finally, RCYs in radiolabeling and the CARs required to meet the RCY criteria for clinical trials of ^89^Zr-DFO-mAb were also evaluated using equations developed from the Zr-DFO complex model refined in this study.

## 2. Results

### 2.1. Mass Spectrometry

**Figure 2:**
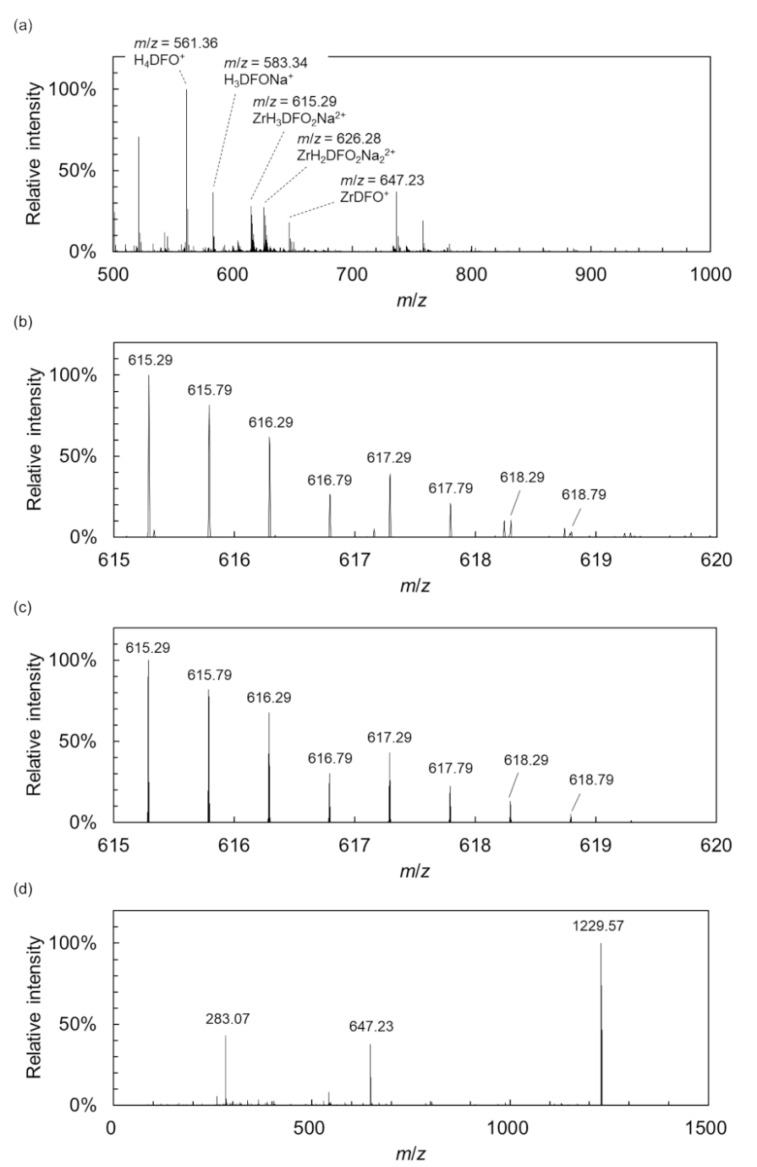
Mass spectra of Zr-DFO complex by Nano-ESI-Q-MS. (**a**) Entire spectrum from *m*/*z* = 500 to 1000. (**b**) Enlarged view of the mass spectra at the range from *m*/*z* = 615 to 620. (**c**) Isotope distribution of ZrH_3_DFO_2_Na^2+^ species. (**d**) Nano-ESI-Q-MS/MS spectra from the precursor ion of *m*/*z* = 1229.571. A disassembled peak (*m*/*z* = 647.23) was observed.

Mass spectra of Nano-ESI-Q-MS are shown in Figure 2. Two peaks derived from ZrHDFO (*m*/*z* = 324.30 accounted for by H_46_C_25_N_6_O_8_Zr^2+^) and ZrDFO (*m*/*z* = 647.23 accounted for by H_45_C_25_N_6_O_8_Zr^+^) were observed in ESI-Q-MS spectra (data not shown). On the other hand, Nano-ESI-Q-MS revealed mass peaks derived from ZrH_3_DFO_2_Na (*m*/*z* = 615.29) and ZrH_2_DFO_2_Na_2_ (*m/z* = 626.28) (Figure 2a). In addition to the above peaks, ZrH_4_DFO_2_ (*m*/*z* = 604.30), ZrH_2_DFO_2_Na (*m*/*z* = 1229.57 accounted for by H_92_C_50_N_12_O_16_NaZr^+^), and Zr_2_H_4_DFO_3_ (*m*/*z* = 618.61 accounted for by H_139_C_75_N_18_O_24_Zr_2_^3+^) were detected by the Nano-ESI-Q-MS (Figures S2 and S3). An enlarged view of the peaks derived from ZrH_3_DFO_2_Na (*m*/*z* = 615.29) is shown in Figure 2b: the peaks clearly correspond to the theoretical isotope distribution of ZrH_3_DFO_2_Na shown in Figure 2c. The mass peak of *m*/*z* = 1229.57 and the disassembled peak of *m*/*z* = 647.23 were also detected in the MS/MS measurement, as shown in Figure 2d.

### 2.2. Potentiometric Titration

Results of the potentiometric titration of DFO (1–5 mmol/L each) are shown in Figure S4 of the Electronic Supplementary Material.

(2) $$K_{ai}=\frac{\left[H\right]\left[H_{p-1}\mathrm{DFO}\right]}{\left[H_{p}\mathrm{DFO}\right]}\;\;\;\;\;\left(i=5-p,\;1\leq p\leq 4\right)$$

p*K*_a_ values of H*_q_*DFO, defined in Equation (2), were determined from the titration curves and are summarized in Table 1. The p*K*_a_ values obtained in this study showed good agreement with those in the previous report [15,16,17,18,19].

(3) $$\{\begin{array}{c} {\left[\mathrm{Zr}\right]}_{T}=\sum^{\;}\left(p\beta_{pqr}{\left[\mathrm{Zr}\right]}^{p}{\left[H\right]}^{q}{\left[\mathrm{DFO}\right]}^{r}+\beta_{\mathrm{Zr}{\left(\mathrm{OH}\right)}_{s}}\left[\mathrm{Zr}\right]{\left[\mathrm{OH}\right]}^{s}\right)\\ {\left[\mathrm{DFO}\right]}_{T}=\sum^{\;}\left(r\beta_{pqr}{\left[\mathrm{Zr}\right]}^{p}{\left[H\right]}^{q}{\left[\mathrm{DFO}\right]}^{r}\right)\\ \sum^{\;}\left\{\left(4p+q-3r\right)\beta_{pqr}{\left[\mathrm{Zr}\right]}^{p}{\left[H\right]}^{q}{\left[\mathrm{DFO}\right]}^{r}+\left(4-s\right)\beta_{\mathrm{Zr}{\left(\mathrm{OH}\right)}_{s}}\left[\mathrm{Zr}\right]{\left[\mathrm{OH}\right]}^{s}\right\}+\left[H\right]-\left[\mathrm{OH}\right]+\left[\mathrm{Na}\right]=0 \end{array}$$

**Figure 3:**
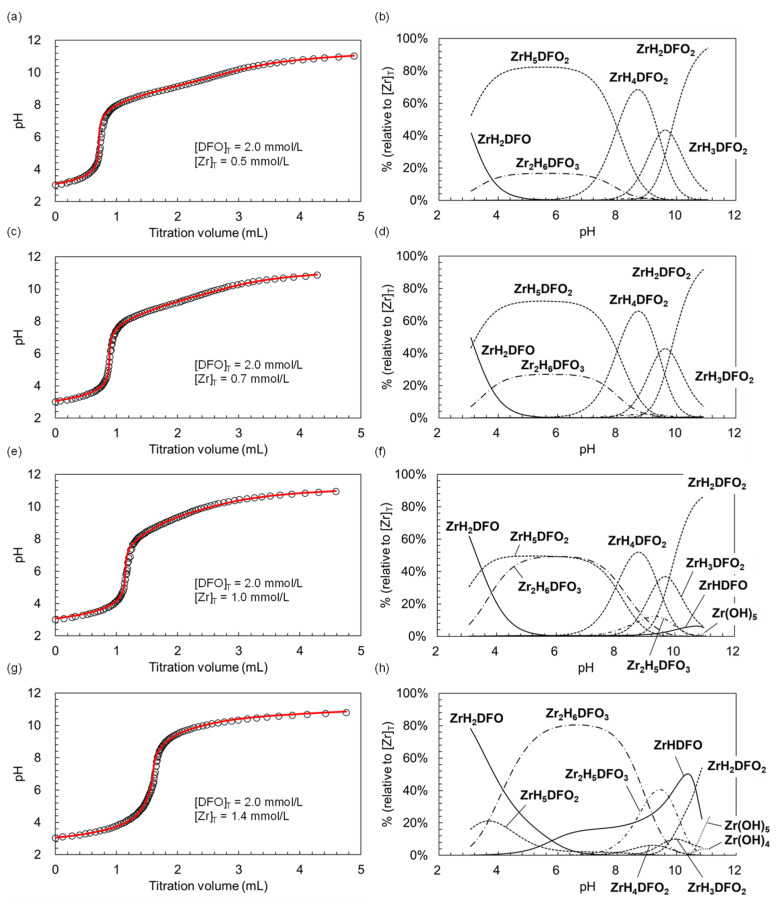
Titration curves and speciation curves of Zr-DFO complex in the co-presence of DFO ([DFO]_T_ = 2.0 mmol/L) and (**a**,**b**) [Zr]_T_ = 0.5 mmol/L (DFO/Zr ratio = 4.0); (**c**,**d**) [Zr]_T_ = 0.7 mmol/L (DFO/Zr = 2.85); (**e**,**f**) [Zr]_T_ = 1.0 mmol/L (DFO/Zr = 2.0); and (**g**,**h**) [Zr]_T_ = 1.4 mmol/L (DFO/Zr = 1.43). Each circle in the titration curve shows an experimental value. The solid red line shows the theoretical curve calculated from the stability constants determined in the potentiometric titration. Speciation curves were generated from Equation (2). All titrations were performed at 25 ℃ in ion strength of 0.15 mol/L NaCl.

Results of the potentiometric titration studies for the co-existence of DFO and Zr are shown in Figure 3a,c,e,g. Plateaus were observed at the range of pH 8.5–9.5 only when the ratio of DFO/Zr was over 2 ([DFO]_T_ = 2.0 mmol/L, [Zr]_T_ = 0–0.7 mmol/L). On the other hand, the plateau at the same pH range became smaller or was not observed when Zr/DFO ratio ≤ 2.0 ([DFO]_T_ = 2.0 mmol/L, [Zr]_T_ = 1.0–1.4 mmol/L). In order to determine stability constants, the simultaneous equations below were solved for each [Na], which is equivalent to the injection volume of NaOH aqueous solution (Equation (3)).

Stability constants of Zr(OH)*_s_* were adopted from the published results [20]. It is noted that formation of ZrCl*_n_* was ignored due to low complexation ability of Cl^−^ to Zr^4+^ ion [21]. The stability constants obtained by fitting the Equation (3) to the titration curves are summarized in Table 2. The stability constants of ZrHDFO (log*β*_111_), ZrH_5_DFO_2_ (log*β*_152_), and Zr_2_H_6_DFO_3_ (log*β*_263_) were 49.1 ± 0.3, 93.3 ± 0.1, and 147.7 ± 0.1, respectively. Titration curves numerically reproduced from the abovementioned stability constants are shown in the solid-line curves of Figure 3a,c,e,g. Each reproduced titration curve was well fitted to the experimental curves in all cases. Speciation curves of the Zr-DFO complex under each condition are shown in Figure 3b,d,f,h.

### 2.3. Competition Studies

**Figure 4:**
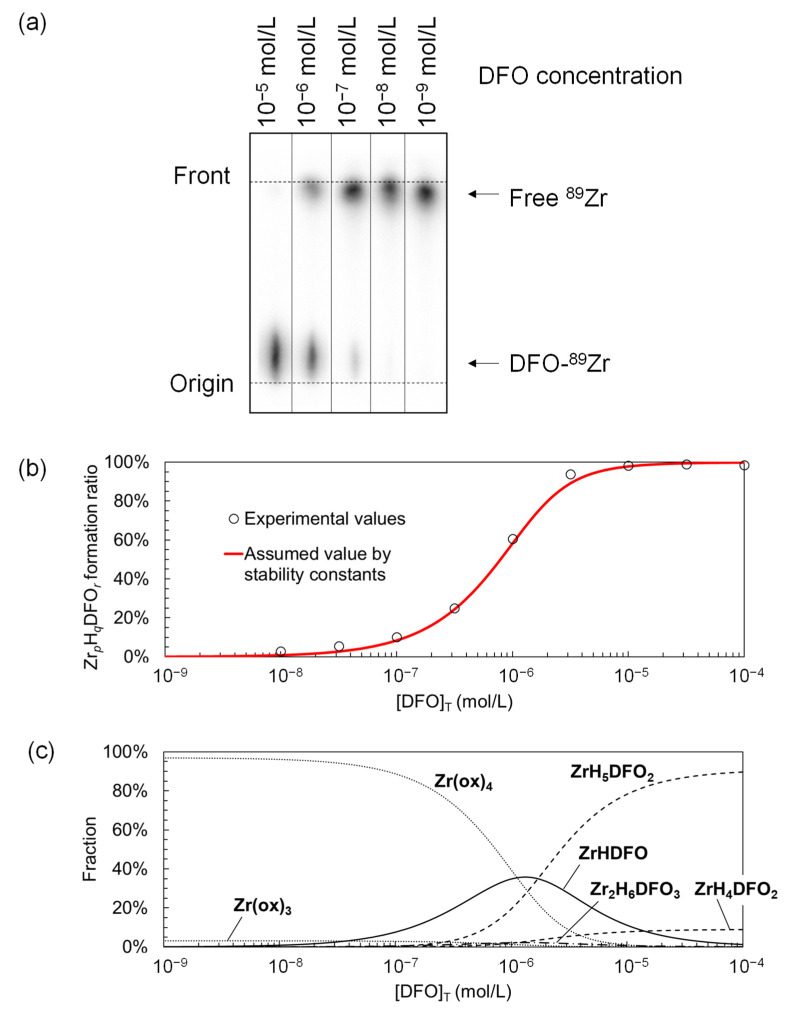
Results of the competition titration. (**a**) ITLC images of the competitive ^89^Zr titration of DFO (10^−9^–10^–5^ mol/L) vs oxalate (0.1 mol/L). Free ^89^Zr was distributed at R*_f_* = 0.8–1.0; ^89^Zr-DFO at R*_f_* = 0.2–0.3. (**b**) Titration curves of Zr*_p_*H*_q_*DFO*_r_* formation ratio against the DFO concentration. The formation ratio is shown as approximately equal to the radioactivity of ^89^Zr-DFO per total radioactivity of ^89^Zr. The open circles show experimental values of the competitive titration, and the red solid line indicates the theoretical curve assumed from the Equation (2) using the calculated stability constants. (**c**) Speciation curves of the Zr*_p_*H*_q_*DFO*_r_*, Zr(OH)*_s_*, and Zr(ox)*_t_* species in the system of DFO (10^–9^–10^–4^ mol/L) oxalate (0.1 mol/L) at pH 7.0. Calculated from the stability constants obtained in this study.

ITLC images of the competitive titration in the presence of various concentrations of Y are shown in Figure S5. Radioactive spots at the retention factor (R*_f_*) of 0.1–0.3 were derived from ^89^Zr-DFO, and those around the front of the TLC were derived from free ^89^Zr. The radiochemical yield of ^89^Zr-DFO was over 95% in all cases. ITLC images of the ^89^Zr distribution in the presence of various concentration of DFO (10^−9^–10^−5^ mol/L) and oxalate (0.1 mol/L) are shown in Figure 4a. The intensity of the ^89^Zr-DFO decreased as the concentration of DFO was diluted. Plots of experimental values are shown in Figure 4b. RCYs of ^89^Zr-DFO obtained in Figure 4a were equivalent to the total amounts of Zr*_p_*H*_q_*DFO*_r_* species in this case. The red solid line shows the simulation curve assumed from the stability constants obtained in this study, which was in turn well fitted to the experimental values, shown as open circles. The speciation curves were also simulated from the modified Equation (4) to consider Zr-oxalate species, Zr(ox)*_t_* (1 ≤ *t* ≤ 4). 

(4) $$\left\{ \begin{array}{c} {\left[\mathrm{Zr}\right]}_{T}=\sum^{\;}\left(p\beta_{\mathrm{pqr}}{\left[\mathrm{Zr}\right]}^{p}{\left[H\right]}^{q}{\left[\mathrm{DFO}\right]}^{r}+\beta_{\mathrm{Zr}{\left(\mathrm{OH}\right)}_{s}}\left[\mathrm{Zr}\right]{\left[\mathrm{OH}\right]}^{s}+\beta_{\mathrm{Zr}{\left(\mathrm{ox}\right)}_{t}}\left[\mathrm{Zr}\right]{\left[\mathrm{ox}\right]}^{t}\right)\\ {\left[\mathrm{DFO}\right]}_{T}=\sum^{\;}\left(r\beta_{pqr}{\left[\mathrm{Zr}\right]}^{p}{\left[H\right]}^{q}{\left[\mathrm{DFO}\right]}^{r}\right)\\ {\left[\mathrm{ox}\right]}_{T}=\left[\mathrm{ox}\right]+\frac{\left[H\right]\left[\mathrm{ox}\right]}{K_{a2,\mathrm{ox}}}+\frac{{\left[H\right]}^{2}\left[\mathrm{ox}\right]}{K_{a1,\mathrm{ox}}K_{a2,\mathrm{ox}}}+\sum^{\;}\left(t\beta_{\mathrm{Zr}{\left(\mathrm{ox}\right)}_{t}}\left[\mathrm{Zr}\right]{\left[\mathrm{ox}\right]}^{t}\right)\\ \sum_{\;}\begin{array}{c} \begin{array}{c} \left\{\left(4p+q-3r\right)\beta_{pqr}{\left[\mathrm{Zr}\right]}^{p}{\left[H\right]}^{q}{\left[\mathrm{DFO}\right]}^{r}+\left(4-s\right)\beta_{\mathrm{Zr}{\left(\mathrm{OH}\right)}_{s}}\left[\mathrm{Zr}\right]{\left[\mathrm{OH}\right]}^{s}+\left(4-2t\right)\beta_{\mathrm{Zr}{\left(\mathrm{ox}\right)}_{t}}\left[\mathrm{Zr}\right]{\left[\mathrm{ox}\right]}^{t}\right\}+\\ \left[H\right]-\left[\mathrm{OH}\right]+\left[\mathrm{Na}\right]-2\left[\mathrm{ox}\right]-\frac{\left[H\right]\left[\mathrm{ox}\right]}{K_{a2,\mathrm{ox}}}=0 \end{array} \end{array} \end{array}\right.$$

The acid dissociation constants of oxalic acid (*K*_a1,ox_ = 10^−1.28^ and *K*_a2,ox_ = 10^−3.65^) and stability constants of Zr(ox)*_t_* were adopted from previously published results [13]. Speciation curves at pH 7.0 taking into account the existence of Zr-DFO (Zr*_p_*H*_q_*DFO*_r_*) and Zr-oxalate (Zr(ox)*_t_*) are presented in Figure 4c. This study showed that Zr-oxalate, Zr(ox)_3_ and Zr(ox)_4_, mainly exist at low concentration of DFO and Zr-DFO complex, while ZrHDFO and ZrH_5_DFO_2_ are dominant at high concentrations of DFO. On the other hand, zirconium hydroxide (Zr(OH)*_s_*) did not seem to be present in this system.

### 2.4. Estimation of RCYs and CARs

We formulated equations based on the following conditions: the speciation curves indicated that both ZrH*_q_*DFO_2_ and Zr_2_H*_q_*DFO_3_ species existed and that Zr(ox)_4_ was dominant (Figure 4c). However, the formation of ZrH*_q_*(DFO-mAb)_2_ bis-complex may be negligible due to steric hindrance between antibodies in the case of radiolabeling to DFO-mAb [22]. The radiolabeling condition is therefore simply modelled as competition between ZrHDFO and Zr(ox)_4_. Because the p*K*_a_ values of DFO and oxalate indicated that DFO is a fully protonated form (H_4_DFO) and most of the oxalate is in the deprotonated form (ox) at pH 7.0, the DFO and Zr(ox)_4_ concentrations can be therefore defined as shown in Equations (5) and (6), respectively.

(5) $$\left[\mathrm{Zr}{\left(\mathrm{ox}\right)}_{4}\right]≅\beta_{\mathrm{Zr}{\left(\mathrm{ox}\right)}_{4}}\left[\mathrm{Zr}\right]{\left[\mathrm{ox}\right]}_{T}{}^{4}$$

(6) $$\left[\mathrm{ZrHDFO}\right]≅\beta_{111}K_{a1}K_{a2}K_{a3}K_{a4}\frac{\left[\mathrm{Zr}\right]{\left[\mathrm{DFO}\right]}_{T}}{{\left[H\right]}^{3}}$$

Therefore, the RCY of ^89^Zr-DFO-mAb ([ZrHDFO]/[Zr]_T_) can be approximately expressed as Equation (7).

(7) $$\frac{\left[\mathrm{ZrHDFO}\right]}{{\left[\mathrm{Zr}\right]}_{T}}≅\frac{1}{1+\left(\beta_{\mathrm{Zr}{\left(\mathrm{ox}\right)}_{4}}{\left[H\right]}^{3}{\left[\mathrm{ox}\right]}_{T}{}^{4}/\beta_{111}K_{a1}K_{a2}K_{a3}K_{a4}{\left[\mathrm{DFO}\right]}_{T}\right)}$$

Furthermore, CAR can be estimated from Equation (6) by substituting the following equation for [DFO]_T_.

(8) $$\left[\mathrm{DFO}-\mathrm{mAb}\right]\times \mathrm{CAR}={\left[\mathrm{DFO}\right]}_{T}$$

Using these equations, the RCYs were estimated under the conditions reported previously [14]. The RCY of ^89^Zr-DFO-mAb estimated by Equation (6) under the typical radiolabeling conditions ([ox]_T_ = 0.1 mol/L, [DFO-mAb] = 0.5 mg/mL, MW_DFO-mAb_ = 150 kDa, CAR = 1.5) was 86%. The RCY of ^89^Zr labeling in the presence of lower oxalate concentration (3.0 × 10^−5^ mol/L) than that of the standard protocol (1.0 × 10^−4^ mol/L) was >97% [23]. Calculation by Equation (6) demonstrated that the CAR with 97% of RCY was estimated to be 3.4 in the presence of 1 mg/mL of mAb and 0.1 mol/L of oxalate. On the other hand, the CAR with the same RCY was 0.3 when the oxalate concentration is 0.05 mol/L.

## 3. Discussion

Coordination chemistry plays significant roles in the design and evaluation of radiopharmaceuticals. Stability constants directly contribute to the binding strengths between radiometals and BFCs, which are in turn closely related to the in-vivo stability and consequently the pharmacokinetics, i.e., biodistribution and metabolism, of the radiometal-labeled radiopharmaceuticals. With further development of informatics on drug design and pharmacokinetics, the significance of thermodynamic parameters in the design of radiometal-labeled radiopharmaceuticals will increase. As interest in ^89^Zr has rapidly increased in recent years, numerous ^89^Zr-DFO-mAbs have been developed as ^89^Zr-immune-PET radiopharmaceuticals, including under clinical uses, such as ^89^Zr-DFO-atezolizumab [24]. Other BFCs containing no hydroxamate moiety, such as DOTA, have also been tested, but DFO remains the typical BFC for ^89^Zr labeling because of its high reactivity at room temperature. However, despite the interest in ^89^Zr-DFO-mAb, only a few studies have reported the stability constants of the Zr-DFO complexes [11,12]. Additionally, the reported stability constants seem to be inaccurate and the speciation of the Zr-DFO complex incomplete due to abovementioned reasons relating to the prediction of RCY. These backgrounds motivated us to re-evaluate the coordination chemistry of the Zr-DFO complex in this study.

In the present study, the nano-ESI-Q-MS spectroscopy successfully detected the mass peaks of ZrH_2_DFO_2_Na, ZrH_3_DFO_2_Na, and ZrH_4_DFO_2_ as well as those previously detected ZrDFO, ZrHDFO, and Zr_2_H_4_DFO_3_ species [11]. These MS studies showed the existence of ZrH*_q_*DFO_2_ species and are the first to obtain direct evidence of the unknown species. In addition, the plateaus at pH 8.5–9.5 observed in the potentiometric titration in the co-presence of DFO and Zr also inferred the presence of ZrH*_q_*DFO_2_ species because of the following reasons; the plateaus at pH 8.5–9.5 was originated from deprotonation of the hydroxamic acids of the non-complexed H*_q_*DFO. The non-complexed DFO existed when DFO/Zr > 2 (Figure 3a,c), which therefore caused the observation of the plateaus. On the other hand, ZrH*_q_*DFO_2_ species were formed, and the non-complexed DFO was negligible when (1 <) DFO/Zr ≤ 2 (Figure 3e,g), which induced the disappearance of the plateaus.

We recalculated the stability constants by taking three species, ZrH*_q_*DFO, ZrH*_q_*DFO_2_, and Zr_2_H*_q_*DFO_3_, into consideration. Consequently, the stability constant of ZrHDFO (log*β*_111_ = 49.1 ± 0.3) was much larger than the previous result (log*β*_111_ = 36.1) and the largest among the *β*_111_ values of the DFO metal complexes [9,15,19,25,26,27]. It should be noted that the simulation curves (solid red lines) obtained from the newly obtained stability constants showed excellent agreement with the experimental titration curves (open circles) in the potentiometric titration (Figure 4), which clearly demonstrated the correctness of our re-evaluated DFO-Zr complex model and its stability constants. It is most likely that both residual yttrium of the target material in the ^89^Zr production process and the oxalate eluent in the ^89^Zr purification process could inhibit the formation of the ^89^Zr-DFO complex because DFO can bind to either of them. Thus, we performed competition titration studies. The competition titration of Zr vs Y to DFO eliminated the concern about Y, as even excess amounts of Y (even Y/Zr ≥ 10^4^ or Y/DFO ≥ 10^2^) did not inhibit the complexation of Zr-DFO. The amount of Y was about ~10^−5^ mol/L in the purified ^89^Zr solution [28]. Considering that purified ^89^Zr solution is diluted 10-fold in radiolabeling reaction, the concentration of Y in radiolabeling reaction is estimated to be about ~10^−6^ mol/L. The competition titration results clearly revealed that remaining Y at the concentration of 10^−6^ mol/L does not inhibit the complexation of Zr-DFO. These results make sense because the YHDFO complex (log *β*_111_ = 16.9) is much less stable than ZrHDFO (log *β*_111_ = 49.1) [19]. On the other hand, the competition titration (DFO vs oxalate to Zr) also demonstrated that the formation of ^89^Zr-DFO and ^89^Zr-oxalate were in competition, as expected. To the best of our knowledge, this is the first study to experimentally show that oxalate inhibits the formation of Zr-DFO complexes. The Zr*_p_*H*_q_*DFO*_r_* formation ratio was assumed by Equation (2) and by the stability constants obtained in this study and is indicated by the solid red line in Figure 4b. Consequently, the assumed values were well fitted to the experimental values indicated by the open circles, which are supportive of the accuracy of the newly obtained stability constants.

As described before, the stability constant obtained from carefully considered complex model can predict the radiochemical yield (RCY) and the optimal chelator-to-antibody ratio (CAR). CAR is now recognized as an especially significant parameter for the evaluation of the immunoreactivity of radiolabeled compounds; the set of optimal CARs will both show high RCY values in the radiolabeling process and maintain a high immunoreactivity. It is known that a high CAR is favorable for achieving a high RCY but that a radiolabeled antibody with high CAR decreases its immunoreactivity because some binding sites to the target-molecule are bound to BFC groups instead. In fact, a recent study reported that ^89^Zr-DFO-mAb with a high CAR showed low tumor uptake [29]. We therefore used the stability constants obtained in this study to estimate the RCY in the radiolabeling of ^89^Zr-DFO-mAb and determine the CAR that would satisfy certain criteria of RCY. The RCY of ^89^Zr-DFO-mAb estimated by Equation (6) under the typical radiolabeling conditions (86%) and the lower oxalate concentration (>99%) were identical to the experimental values (87% [14] and >97% [23]). These results clearly indicated that the experimental RCYs were explained well by the Equation (6). Even though log*β*_111_ determined in this study much differs with previous study [11,12], the good agreement between experimental and theoretical RCY indicates the accuracy of our stability constants.

We then tried to estimate CARs to satisfy certain criteria of RCY. We set the criteria for RCY in this study as 97% because the IAEA quality control guideline recommended a radiochemical purity of >95% for ^89^Zr-DFO-trastuzumab for its clinical trials [30]. Calculation by Equation (6) demonstrated that the CAR with 97% of RCY was estimated to be 3.4 in the presence of 1 mg/mL of mAb and 0.1 mol/L of oxalate. On the other hand, the CAR with the same RCY is lower (0.3) when the oxalate concentration is 0.05 mol/L. These results demonstrated that a lower concentration of oxalate is desirable to minimize the CAR when meeting the RCY criteria since a low CAR is generally preferable to maintain the immunoreactivity of the desired radiolabeled antibodies, as described above. Much experimental effort is still necessary to optimize CAR because the number of DFO-mAbs with different CARs are prepared and tested under different conditions in terms of both RCY and immunoreactivity. However, the reported approach enables us to narrow down the conditions from the standpoint of RCY, so it will help to find the optimal CAR for ^89^Zr-DFO-antibodies with less effort. As a result, DFO-mAb with optimal CAR will be utilized to the manufactural preparation of ^89^Zr-DFO-mAb radiopharmaceuticals for immune-PET. Furthermore, we expect that these findings will greatly contribute to the design of other radiometal labeled radiopharmaceuticals for clinical use.

## 4. Materials and Methods

### 4.1. Chemicals

All aqueous solutions were prepared with distilled water. DFO (Apollo Scientific Ltd., Stockport, UK), 1.0 mol/L NaOH solution (Nacalai Tesque, Kyoto, Japan), NaCl, citric acid (Fujifilm Wako Pure Chemical, Osaka, Japan, guaranteed reagent grade), 1.0 g/L ZrOCl_2_ in 1.0 mol/L HCl solution (Hayashi Pure Chemical Industries, Osaka, Japan), HEPES (Dojindo Molecular Technology, Kumamoto, Japan), and yttrium (Y) foil (Alfa Aesar, Heysham, UK) were used without further purification. 

### 4.2. Mass Spectrometry

In the mass spectrometry experiments, two types of ionization techniques were tried: electrospray ionization (ESI) and nano-ESI. Mass-spectral data on ESI-Q-MS experiments were collected on a Shimadzu LCMS-8040 mass spectrometer, and data on Nano-ESI-Q-MS experiments were collected on a Thermo Scientific™ Q Exactive™ mass spectrometer, Waltham, USA. Each experiment was conducted in positive mode using the flow injection method. The contents of sample solutions were as follows: Zr: 0.5 mmol/L, DFO: 1.0 mmol/L, NaCl: 0.15 mol/L, and HEPES: 0.1 mol/L (pH 7.0). The concentration of Zr was aligned with a previous study (Zr: 1.0 × 10^−3^ mol/L, DFO: 1.0 × 10^−3^ mol/L) [11]. Since the presence of ZrH*_q_*DFO_2_ was inferred from the results of the potentiometric titration experiment, the concentration of Zr was reduced to half. HEPES buffer was used in order to maintain pH of the reactant at 7.0. The solutions were incubated for 1 h at room temperature to equilibrate Zr-DFO system. Sample solutions were diluted 500-fold just before the MS spectrometry measurement. 

### 4.3. Potentiometric Titration

A C-171 automatic titrator (Kyoto Electronics Manufacturing Co., Ltd., Kyoto, Japan) was used for the potentiometric titration studies. The electrode was filled with 3.3 mol/L KCl solution. The initial volume of the reaction solution was 50 mL, and the initial concentration of DFO ([DFO]_T_) and Zr ([Zr]_T_) were adjusted to 1.0–5.0 × 10^−3^ mol and 0.5–1.4 × 10^−3^ mol, respectively. Ionic strength (*I*) was adjusted to 0.15 mol NaCl in all measurements. The pH was adjusted to below 3 before the titration measurement. N_2_ gas was then bubbled through the mixture, which was then vigorously stirred for at least 1 min to drive off dissolved CO_2_ and to allow the solution and the electrode to equilibrate at the 25 °C. Finally, the sample solution was titrated to pH 11 with certified carbonate-free NaOH in dynamic mode, meaning that the amount of 0.1 mol/L NaOH solution added per shot was continuously adjusted by the protocol to achieve an even rate of pH increase. The p*K*_a_ values of H*_q_*DFO and the stability constants β*_pqr_* of Zr-DFO were determined by the non-linear least-squares fitting of titration curves using the Equations (1)–(3). Theoretical pH curves against NaOH amount were reproduced by calculation with Equations (1)–(3) using each combination of p*K*_a_ and β*_pqr_*. The most fitted combination of p*K*_a_ and β*_pqr_* was explored.

### 4.4. Production of ^89^Zr

Production of ^89^Zr was carried out using an AVF cyclotron at Takasaki Ion Accelerators for Advanced Radiation Applications (TIARA) at Takasaki Advanced Radiation Research Institutes, the National Institutes for Quantum and Radiological Science and Technology (QST). ^89^Zr was produced via the ^89^Y(*p*,*n*)^89^Zr reaction, irradiating ^89^Y foil (weight: 0.36 g; size: 10 mm × 10 mm × 0.8 mm) with 20 MeV proton beams accelerated by the AVF cyclotron. The incident energy of the proton beam upon the Y targets was adjusted to 13 MeV by 1.2-mm thick Al degraders in order to prevent the generation of ^88^Zr. The produced radionuclides were characterized by gamma-ray spectrometry by using a high-purity germanium (HPGe) detector coupled to a multichannel analyzer MCA 7700 (SEICO EG&G Co., Ltd., Tokyo, Japan). The radioactivity of ^89^Zr was determined by considering the gamma-ray energy at 909.2 keV. As a result of characterization by gamma-ray spectrometry, only the radiation from ^89^Zr was detected, and the intrinsic gamma rays of ^88^Zr (392.9 keV) were not detected. The generated ^89^Zr activity per irradiation time and beam current was 23.1 MBq/µAh in our experiments. Purified [^89^Zr] chloride (^89^ZrCl_4_) solution was prepared from irradiated Y targets by the procedures described by Holland et al. [31]. Typically, 80–90% of ^89^Zr was recovered from the irradiated target as ^89^ZrCl_4_.

### 4.5. Competition Titration

Competition titration studies were performed with ITLC-SG Chromatography paper (Agilent Technologies, Santa Clara, CA, USA). Zr versus Y competition titration to DFO was conducted as follows. Purified [^89^Zr]ZrCl_4_ solution (typically 0.1–1.0 MBq), nonradioactive ZrCl_4_ solution, yttrium chloride (YCl_3_) solution, HEPES buffer (pH 7.0), and oxalic acid solution were added to DFO solution in a 1.5 mL Eppendorf tube. The final concentrations were DFO 5.0–10 mol/L, Zr 10^−7^ mol/L, Y 10^−8^–10^−3^ mol/L (Y/Zr ratio was 10^−1^–10^4^), HEPES 0.25 mol/L, and oxalate 0.1 mol/L. The final pH of each mixture was maintained at 7.0. Each mixture was incubated for 1 h at room temperature. After incubation, an aliquot of each mixture was spotted on ITLC paper and analyzed using 0.02 mol/L citrate buffer (pH 5.0) as the mobile phase. Competition titration of DFO versus oxalate complexation to Zr was also conducted using a mixture solution of DFO 10^−9^–10^−4^ mol/L, HEPES 0.25 mol/L and oxalate 0.1 mol/L (pH 7.0) without adding nonradioactive Zr or Y. Incubation and ITLC conditions were the same as in the Zr versus Y competition titration.

## 5. Conclusions

In the present study, highly accurate stability constants for the Zr-DFO complexes were successfully determined. This study also demonstrated that the newly obtained stability constants and consideration of the inhibition by the Zr-oxalate complex enabled the equations to accurately calculate the RCYs and the CARs such that they satisfied certain criteria on RCYs. We therefore concluded that our re-evaluated Zr-DFO complex model was a success. Accurate thermodynamic parameters consequently will contribute significantly to provide a theoretical framework for quantitative calculation of RCY from CAR, antibody, and oxalate concentrations in the process of developing new Zr-DFO mAb for clinical application, and as a result, DFO-mAb with optimal CAR will be used for the preparation of high-quality ^89^Zr-DFO immune-PET tracers.

## Figures and Tables

**Table 1:** The log p*K_ai_* of DFO (1 ≤ *i* ≤ 4) obtained at various ionic strengths *I* (mol/L) of NaCl.

	p*K*_a1_	p*K*_a2_	p*K*_a3_	p*K*_a4_	Ref
0.1	8.4 ± 0.3	9.2 ± 0.1	9.7 ± 0.2	11.0 ± 0.3	[18]
0.15	8.38 ± 0.03	8.96 ± 0.04	9.66 ± 0.08	11.31 ± 0.25	This study
0.15	8.207	8.811	9.381	10.397	[16]
0.60	8.33 ± 0.03	8.96 ± 0.03	9.45 ± 0.05	10.85 ± 0.05	[15]
0.70	8.40 ± 0.01	8.93 ± 0.02	9.58 ± 0.02	10.74 ± 0.09	[19]
1	8.6 ± 0.4	9.2 ± 0.3	9.8 ± 0.2	10.9 ± 0.1	[17]

**Table 2:** The log *β_pqr_* of Zr-DFO complexes determined in this study.

*p*	*q*	*r*	log *β_pqr_*
1	1	1	49.1 ± 0.3
1	2	1	54.9 ± 0.3
1	2	2	66.1 ± 0.1
1	3	2	75.9 ± 0.3
1	4	2	85.3 ± 0.1
1	5	2	93.3 ± 0.1
2	5	3	138.7 ± 0.1
2	6	3	147.7 ± 0.1

## Data Availability

The datasets for this manuscript can be obtained from the corresponding author upon reasonable request.

## Supplementary Materials

The following are available online. Nano ESI MS spectra (ZrDFO^+^, ZrH_2_DFO_2_Na^+^, and Zr_2_H_4_DFO_3_^3+^), titration curves and speciation curves of DFO, and ITLC images of the competitive DFO titration of Zr versus Y are available online. Figure S1: Nano ESI MS spectra of ZrDFO^+^, Figure S2: Nano ESI MS spectra of ZrH2DFO2Na+, Figure S3: Nano ESI MS spectra of Zr_2_H_4_DFO_3_^3+^, Figure S4: Titration curves and speciation curves of DFO ([DFO]_T_ = 1.0–5.0 mmol/L), Figure S5: ITLC images of the competitive DFO titration of Zr (10^−7^ mol/L) versus Y (10^−8^–10^−3^ mol/L)
