# Supplementary material for: Re-Evaluations of Zr-DFO Complex Coordination Chemistry for the Estimation of Radiochemical Yields and Chelator-to-Antibody Ratios of 89Zr Immune-PET Tracers
Source: Molecules. 2021 Aug 17;26(16):4977. doi: 10.3390/molecules26164977 (PMC8401698; doi:10.3390/molecules26164977)
Supplement: Supplementary file 1 [file molecules-26-04977-s001.zip › molecules-1315300-supplementary.pdf]

Re-Evaluations of Zr-DFO Complex Coordination Chemistry for the Estimation of Radiochemical Yields and Chelator-to-Antibody Ratios of ^{89}Zr Immune-PET Tracers

Ryota Imura,[1,2] Hiroyuki Ida, [2] Ichiro Sasaki, [3] Noriko S. Ishioka [3] and Shigeki Watanabe [3,*]

[1] Research Center for Advanced Science and Technology, The University of Tokyo, 4-6-1 Komaba, Meguro-ku, Tokyo 153-8904, Japan; imura-ryota@ric.u-tokyo.ac.jp (R.I.)

[2] JFE Engineering Corporation, 2-1 Suehiro-cho, Tsurumi-ku, Yokohama 230-8611, Japan; ida-hiroyuki@jfe-eng.co.jp (H.I.)

[3] Department of Radiation-Applied Biology Research, Takasaki Advanced Radiation Research Institute, National Institute of Quantum and Radiological Science and Technology (QST), 1233 Watanuki, Takasaki, Gunma 370-1292, JapanJFE Engineering Corporation, sasaki.ichiro@qst.go.jp (I.S.); ishioka.noriko@qst.go.jp (N.S.I); watanabe.shigeki@qst.go.jp (S.W.)

Corresponding author: Shigeki Watanabe (watanabe.shigeki@qst.go.jp)

Supplementary Figure S1.	Nano ESI MS spectra of $ZrDFO^+$.
Supplementary Figure S2.	Nano ESI MS spectra of $ZrH_2DFO_2Na^+$.
Supplementary Figure S3.	Nano ESI MS spectra of $Zr_2H_4DFO_3^{3+}$.
Supplementary Figure S4.	Titration curves and speciation curves of DFO ($[DFO]_T$ = 1.0–5.0 mmol/L)
Supplementary Figure S5.	Figure S5. ITLC images of the competitive DFO titration of Zr (10^{-7} mol/L) versus Y (10^{-8}–10^{-3} mol/L)

(a)

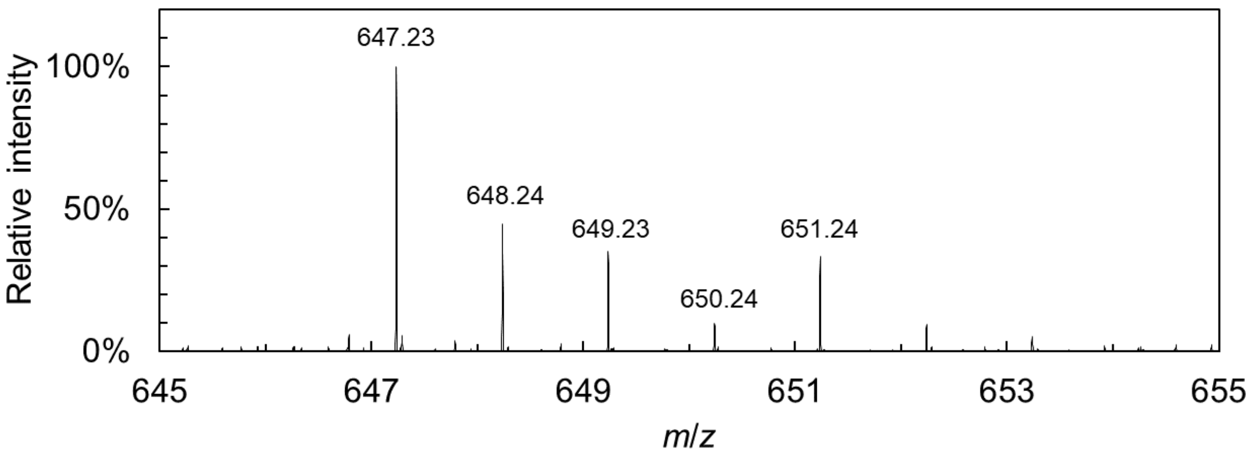

(b)

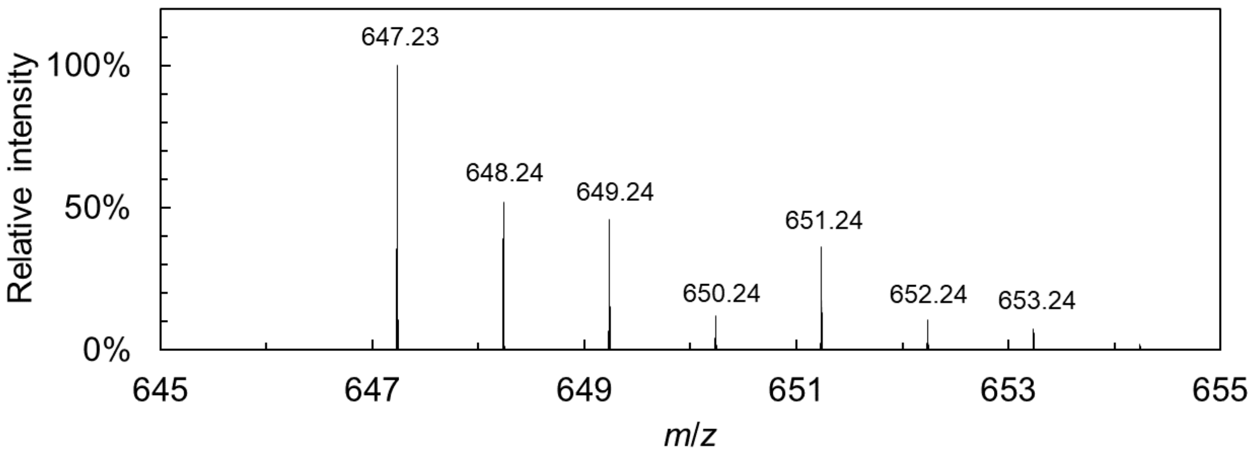

Figure S1. Nano-ESI-MS spectra of $ZrDFO^+$. (a) Mass spectra in the range of *m*/*z* between 645 and 655.; (b) Simulation results of isotope distribution of $ZrDFO^+$.

(a)

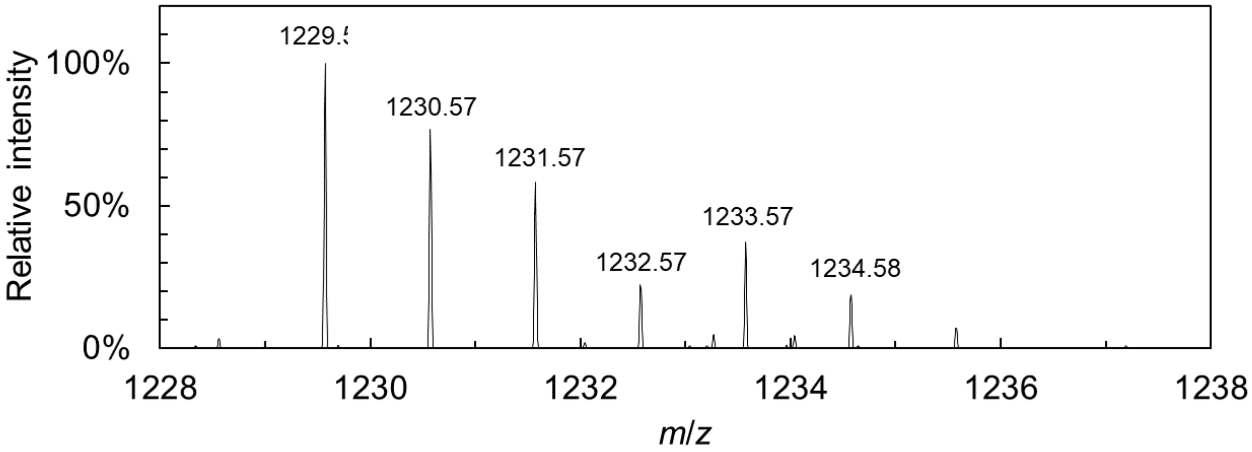

(b) .

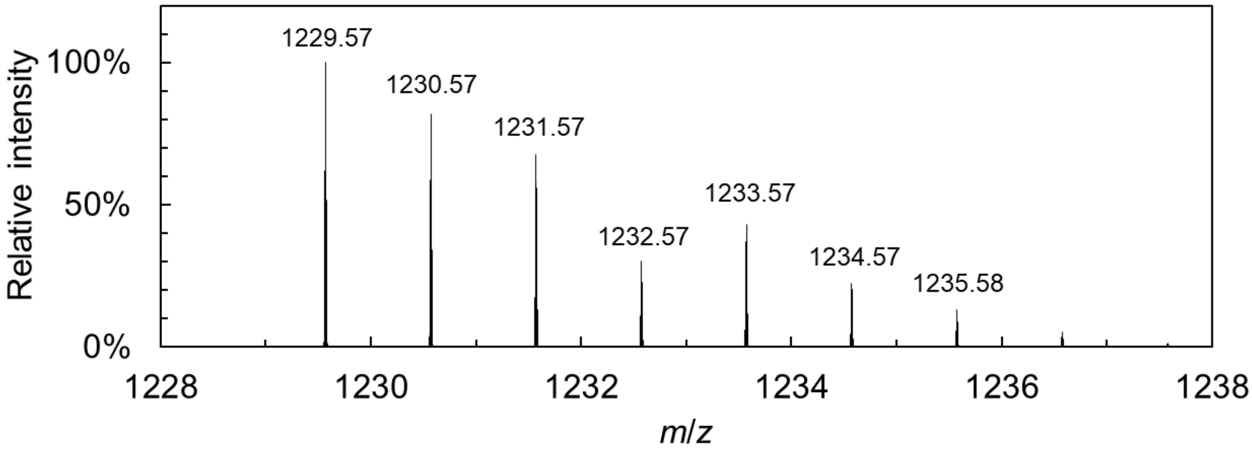

Figure S2. Nano-ESI-MS spectra of $ZrH_2DFO_2Na^+$. (a) Mass spectra in the range of *m*/*z* between 1228 and 1238. (b) Simulation results of isotope distribution of $ZrH_2DFO_2Na^+$

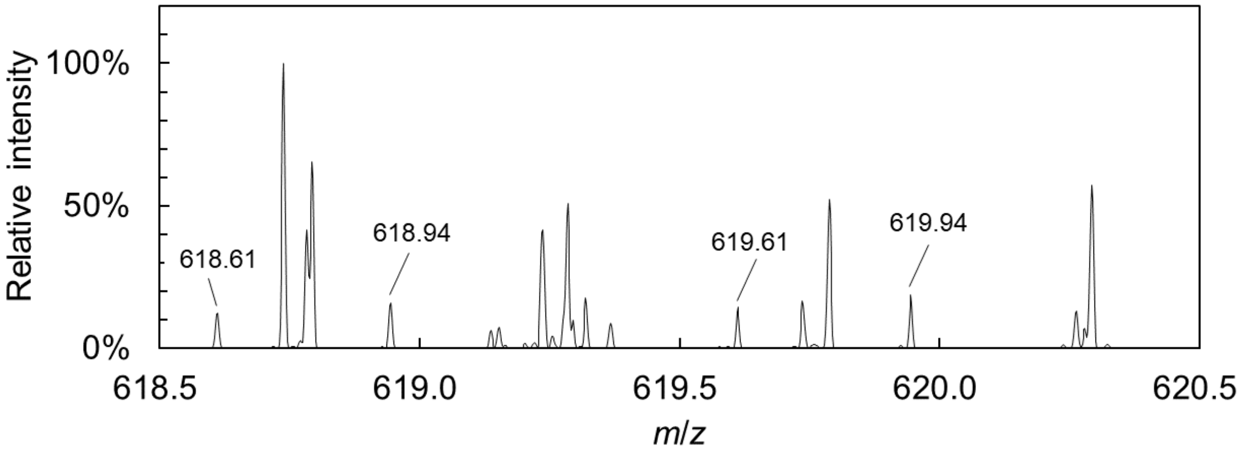

(a) Mass spectra in the range of *m*/*z* between 618.5 and 620.5. Mass peaks originated from $Zr_2H_4DFO_3^{3+}$ were detected in this *m*/*z* region.

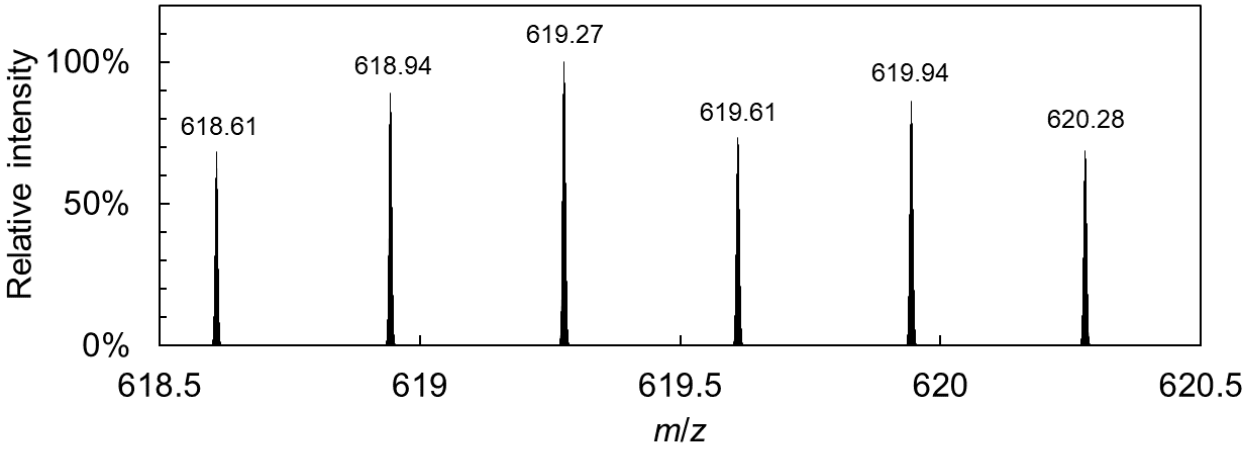

(b) Simulation results of isotope distribution of $Zr_2H_4DFO_3^{3+}$.

Figure S3. Nano-ESI-MS spectra of $Zr_2H_4DFO_3^{3+}$. (a) Mass spectra in the range of *m*/*z* between 1228 and 1238. (b) Simulation results of isotope distribution of $Zr_2H_4DFO_3^{3+}$

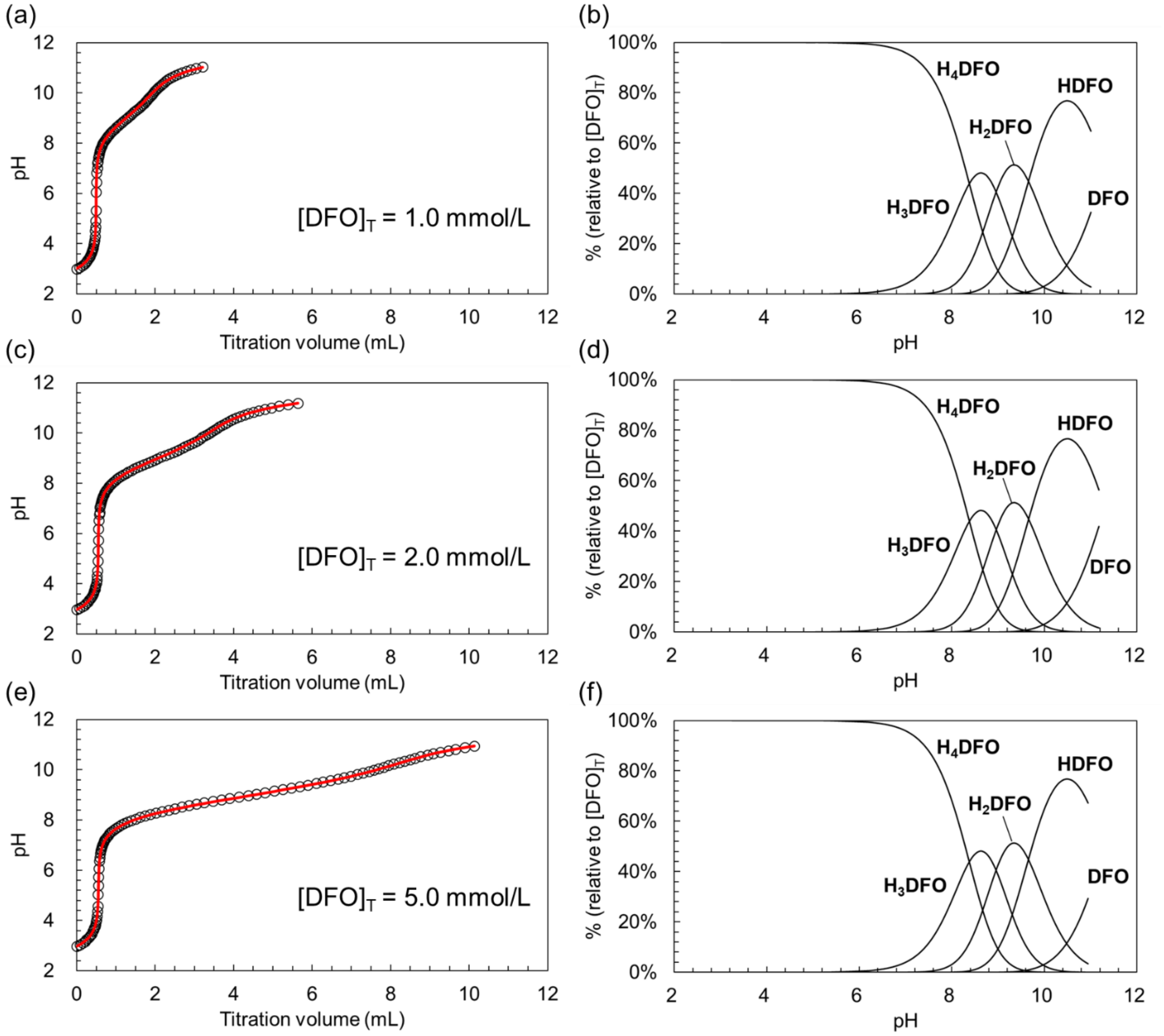

Figure S4. Titration curves and speciation curves of DFO ($[DFO]_T$ = 1.0–5.0 mmol/L). Each circle in the titration curve shows an experimental value. The solid red line shows the theoretical curve calculated from the stability constants determined in the potentiometric titration. Speciation curves were generated from Equation (2).

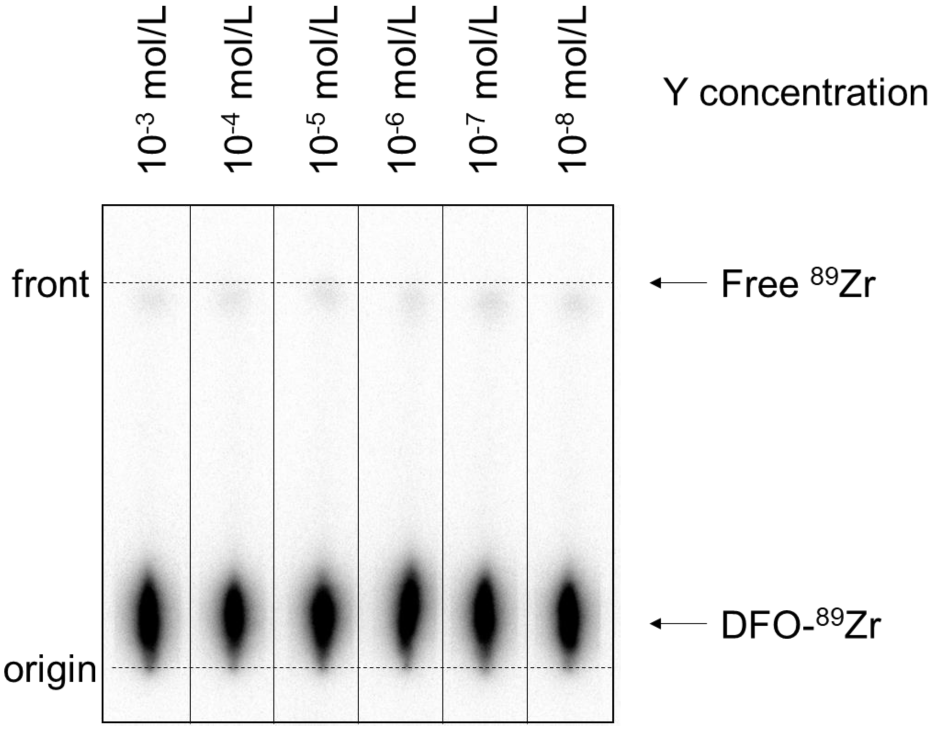

Figure S5. ITLC images of the competitive DFO titration of Zr (10^{-7} mol/L) versus Y (10^{-8}–10^{-3} mol/L). These experiments were performed in the presence of oxalate 0.1 mol/L, and 0.25 mol/L HEPES buffer (pH 7.0).
